# Evolutionary Genomics Provides Insights Into Endangerment and Conservation of a Wild Apple Tree Species, *Malus sieversii*

**DOI:** 10.1111/eva.70048

**Published:** 2024-12-04

**Authors:** Jian Zhang, Fang‐Yuan Zhao, Hong‐Xiang Zhang

**Affiliations:** ^1^ State Key Laboratory of Desert and Oasis Ecology, Key Laboratory of Ecological Safety and Sustainable Development in Arid Lands, Xinjiang Institute of Ecology and Geography Chinese Academy of Sciences Urumqi China; ^2^ Xinjiang Key Laboratory of Conservation and Utilization of Gene Resources Urumqi China; ^3^ Specimen Museum of Xinjiang Institute of Ecology and Geography Chinese Academy of Sciences Urumqi China; ^4^ University of Chinese Academy of Sciences Beijing China

**Keywords:** conservation managements, deleterious mutations, effective population size, evolutionary genomics, *Malus sieversii*

## Abstract

Understanding the evolutionary history of a species is essential for effective conservation management. *Malus sieversii* , a relict broad‐leaf forest tree found in arid Central Asian mountains, has a narrow and fragmented distribution and is classified as an endangered species in China. This species is considered one of the ancestors of the domesticated apple trees. In the present study, we sampled five populations of *M. sieversii* and its wide‐ranging congener *M. baccata* from China. Through deep whole‐genome resequencing, we analyzed the population's genetic diversity, genetic structure, demographic history, fixation of deleterious mutations, and genomic divergence. Our results revealed that *M. baccata* exhibits a higher level of genetic diversity than *M. sieversii* . The effective population size of *M. sieversii* decreased, whereas that of *M. baccata* recovered after the bottleneck effect. In *M. sieversii* , the genetic structure of the Yili region was distinct from that of the Tacheng region. Populations at the rear edge of the Tacheng region showed a stronger fixation of deleterious mutations than those in the Yili region. Genomic divergence indicated that the positively selected genes were associated with physiological processes within the genomic islands between the Yili and Tacheng regions. Based on these findings, we recommend the establishment of two separate conservation units for the Yili and Tacheng lineages to preserve their genetic resources. Given the limited distribution range and high fixation rate of deleterious mutations, urgent protective measures are recommended for the Tacheng lineage.

## 1 Introduction

The endangerment and conservation of species are fundamental concerns in conservation biology. Species are endangered because of their evolutionary history, habitat changes, reproductive characteristics, and human disturbance (Murray et al. 2014; Liu et al. 2015; Cook and Sgro 2017). Relict species characterized by narrow distribution ranges are typically prioritized for conservation efforts. Historically, these species may have had broader distributions under more favorable environmental conditions (Shen et al. 2022; Wang et al. 2023). However, owing to orogenic and paleoclimatic changes, the distribution ranges of relict species have progressively contracted over their evolutionary history. Declines in the effective population size further restricted their distribution to narrow ranges. Thus, investigating the evolutionary history of these relict species is crucial in understanding the mechanisms underlying their endangerment and developing effective conservation strategies.

Genomics provides novel tools to understand the evolutionary history of endangered species and support conservation efforts (Formenti et al. 2022; Schiebelhut et al. 2024). Conservation genomics, facilitated by reference genomes, addresses issues such as inbreeding, deleterious mutations, local adaptation, and genetic rescue among the populations of endangered species (Formenti et al. 2022). For example, when compared to their wider‐ranging congeners, narrow‐ranged species typically exhibit increased genetic drift and fixation of strongly deleterious mutations (Yang et al. 2018; Feng et al. 2024). Genomic resequencing across different populations has been used to assess population vulnerability and adaptive capacity to climate change in temperate forest tree species in East Asia (Sang et al. 2022). This approach identified the most vulnerable populations for conservation prioritization and highlighted numerous candidate genes for forest tree breeding.

In arid Central Asia, relict deciduous broad‐leaf forests are distributed in the middle altitudes of mountains. This specialized habitat provides suitable temperature and precipitation conditions for relict forests, which are currently fragmented across the Central Asian Mountains (Zhang 1973). Species within these forests include *Malus sieversii* , *Prunus cerasifera* , *Juglans regia* , and *Prunus armeniaca* , collectively referred to as wild fruit forests. *M. sieversii* serves as the foundational species for these relict forests and is predominantly found in the Tianshan Mountains of Central Asia, with origins dating back to the Late Miocene (Zhang 1973; Zhang et al. 2021). The fragmented distribution of *M. sieversii* is believed to have been influenced by increasing aridification in the Tianshan Mountains (Zhang et al. 2021). In China, the two main distribution ranges are preserved in the Yili and Tacheng regions. Currently, the *M. sieversii* populations cover approximately 41.5 km^2^ in the Yili region and 9.3 km^2^ in the Tacheng region (Yan and Xu 2010). *M. sieversii* is classified as a second‐ranked conservation priority in the China Plant Red Data Book (Fu 1992). This species is recognized as the ancestor of the domesticated apple trees (Cornille, Gladieux, and Giraud 2013).

Several previous studies have investigated the population genetic diversity and structure of *M. sieversii* (Zhang, Li, and Li 2018; Zhang et al. 2021; Omasheva et al. 2017). Genomic SNP data identified three genetic lineages across the entire distribution range of *M. sieversii* (Zhang et al. 2021). These previous studies suggest significant genetic divergence between the Yili and Tacheng regions among Chinese *M. sieversii* populations. However, previous studies have not determined whether these regions share the same demographical histories. Genetic evidence of divergence between these two regions was not discernible in the genomic landscape. Lastly, it was not demonstrated whether rear‐edge populations in the Tacheng region harbored more deleterious mutations than populations in the widely distributed Yili region.

To address these questions, we sampled five populations of the endangered *M. sieversii* from the Yili and the Tacheng regions. Additionally, we sampled thirteen individuals of a wider‐ranged congener of *M. baccata* . Using deep genome sequencing, our objectives were to (1) investigate the genetic diversity and structure of these *Malus* samples, (2) elucidate their evolutionary history and genetic divergence, and (3) test the hypothesis that species with narrow geographical ranges may accumulate a higher prevalence of strongly deleterious mutations.

## 2 Materials and Methods

### 2.1 Plant Sampling, DNA Extraction, Genomic Resequencing, and SNP Calling

**FIGURE 1:**
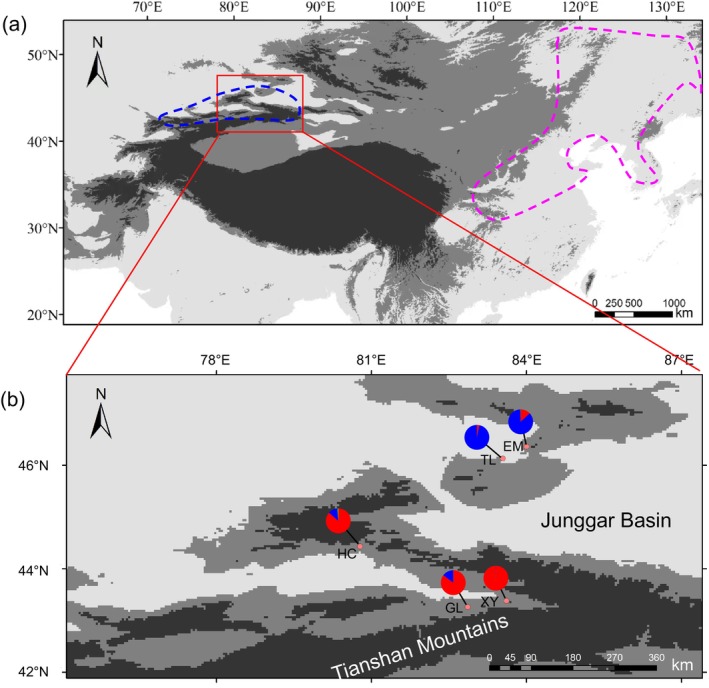
(a) Distribution ranges of *Malus sieversii* (blue dashed line) and *M. baccata* (pink dashed line); (b) Geographic distribution of the ADMIXTURE clusters for the 5 populations of *M. sieversii* at *K* = 2.

We devised a sampling strategy to investigate the genetic diversity of *M. sieversii* and *M. baccata* . For *M. sieversii* , we selected a total of 40 individuals from five different populations: three from the Yili region (HC, GL, and XY) and two from the Tacheng region (EM and TL) (Figure 1). Eight individuals were randomly sampled from each population, ensuring that each sample was collected more than 50 m apart from others to minimize genetic relatedness. Additionally, 13 fresh leaf samples were collected from *M. baccata* . In the laboratory, total genomic DNA was extracted from leaf materials using the DNeasy Plant Mini Kit (Qiagen, Hilden, Germany), following the manufacturer's instructions.

To analyze the genomic structures of the samples from *M. sieversii* and *M. baccata* , we employed a whole‐genome sequencing approach to construct libraries with varying insert sizes. This method involves the random fragmentation of genomic DNA, generating fragments of diverse lengths to ensure comprehensive coverage of the entire genome. We used the Illumina Novaseq platform for paired‐end sequencing, which utilizes second‐generation sequencing technology known for its high throughput and accuracy. This technology produces abundant sequencing data suitable for subsequent genome assembly and analysis.

Throughout the experiment, we rigorously monitored sample quality. Initially, each sample underwent testing for DNA purity and concentration to meet sequencing requirements. Subsequently, the qualified DNA fragments were integrated into sequencing libraries. The libraries were sequenced using the Illumina Novaseq platform to generate a substantial amount of read data. Following sequencing, we promptly assessed the quality of the post‐sequencing data using FastQC tools (v0.11.8) (Wingett and Andrews 2018). Through FastQC analysis, we ensured the high quality and reliability of the sequence data used for subsequent variant detection, mutation detection, and functional analysis. For all subsequent analyses, we aligned the reads to the *M. domestica* genome reference obtained from (https://www.ncbi.nlm.nih.gov/datasets/genome/GCF_002114115.1/). To investigate the potential genetic introgression of *M. domestica* into these *Malus* samples, we downloaded 13 *M. domestica* samples from the NCBI database.

Following quality control of the sequencing data with FastQC, we employed the BWA software (v0.7.17) (Li and Durbin 2010), specifically the Burrows–Wheeler Aligner, to align the high‐quality data with reference genomes. Post‐alignment, Picard software (v2.18.29) (http://broadinstitute.github.io/picard/) was used with default parameters to tag polymerase chain reaction (PCR) duplicates. These duplicates arise from random biases during PCR amplification and may introduce bias into variant detection. Tagging PCR duplicates allows appropriate processing or exclusion in subsequent analyses, thereby minimizing their impact on variant detection outcomes. Finally, variant detection was performed using Genome Analysis Toolkit (GATK) software (v4.1.4.1) (McKenna et al. 2010). Identified SNPs were filtered using criteria “*QD < 2.0 || MQ < 40.0 || QUAL < 30.0 || FS > 60.0 || SOR > 4.0 || MQRankSum < −12.5 || ReadPosRankSum < −8.0*” and subsequently filtered further to exclude: (1) SNPs lacking genotypic data (no missing genotypes allowed) and (2) SNPs with minor allele frequencies exceeding 0.05.

### 2.2 Genetic Structure Analyses

To gain a comprehensive understanding of the genetic structure and population relationships within *M. sieversii* and *M. baccata* populations, we employed various bioinformatics methods to analyze filtered single nucleotide polymorphism (SNP) data. Initially, we utilized PLINK (v1.90b6.21) (Purcell et al. 2007) to prune the SNP dataset, thereby mitigating linkage disequilibrium effects, prior to conducting a Bayesian clustering analysis using ADMIXTURE software (v1.3.0) (Alexander, Novembre, and Lange 2009). This preprocessing step was essential for enhancing the independence of the genetic markers and ensuring the accuracy of the subsequent population structure analysis and ancestral component inference. For the Bayesian clustering analysis, we explored a range of genetic clusters (*K*) from 2 to 10, selecting the *K* value associated with the lowest cross‐validation error to delineate the optimal population structure. To confirm the absence of admixture from *M. domestica* in our samples, we also downloaded 13 *M. domestica* samples from different regions via NCBI and subjected them to the same analysis. Additionally, to validate and visualize genetic relationships among populations, we performed principal component analysis (PCA) on the same SNP dataset using the PLINK software (Purcell et al. 2007). Furthermore, to elucidate the evolutionary relationships among the populations, we conducted maximum likelihood (ML) tree analysis using IQ‐tree (v2.0.6) (Bui Quang et al. 2020), which facilitated the construction of phylogenetic trees.

Following the determination of population structure and genetic relationships, we utilized VCFTOOLS (v0.1.16) (Danecek et al. 2011) software to statistically analyze polymorphism data in nonoverlapping 100 kb windows for each species and within‐species lineage. This analysis encompassed nucleotide diversity (*θ*_*π*_), Watterson's estimator (*θ*_*W*_), and Tajima's D, crucial metrics for assessing the genetic diversity and selection pressures. Additionally, we calculated the genome‐wide individual heterozygosity (*H*) and inbreeding coefficient (*F*_IS_) between populations using VCFTOOLS (Danecek et al. 2011) and PLINK software (Purcell et al. 2007).

### 2.3 Demographical History Analysis

To explore the population history dynamics of *M. baccata* and *M. sieversii* , we employed the Pairwise Sequentially Markovian Coalescent (PSMC) method (v0.6.5) (Schiffels and Durbin 2014) to estimate changes in effective population size (Ne) over time using whole‐genome data. Two individuals per population were selected to represent the genetic characteristics of each lineage. Input data for PSMC were prepared using the BCFTools (v1.9) (Danecek et al. 2021) software to generate chromosome consensus sequences. These sequences included genomic and variant information from two selected individuals.

We established the running parameters for PSMC as follows: the number of iterations was set to 25, allowing PSMC to perform 25 iterations within each time interval to optimize the population size estimation. We also specified five random starting points to conduct multiple independent runs and reduce the impact of initial conditions on the results. For the model parameters of PSMC, we defined “4 + 25 * 2 + 4 + 6,” which determines the number and duration of blocks in the model, influencing PSMC's sensitivity to changes in population history. Additionally, based on previous studies and evolutionary rate estimates, we fixed the base mutation rate at 3.9 × 10^−8^ per site per generation (Sun et al. 2020). This mutation rate is crucial for assessing population history dynamics in PSMC. Finally, we set the generation time to 8 years according to species‐specific data (Yamagishi, Kishigami, and Yoshikawa 2014), enabling PSMC to interpret genomic data variations as fluctuations in population size over time.

### 2.4 Mutation Load Analysis

To compare the whole‐genome mutation loads between *M. sieversii* and *M. baccata* , our objective was to identify mutations that are potentially detrimental to gene function. We followed the following structured analytical approach: Initially, we used the *M. domestica* genome reference to construct the SNPEFF database (v4.3t) (Cingolani et al. 2012). SNPEFF was used to classify SNPs in both species based on their position and context, distinguishing between synonymous, non‐synonymous, and loss‐of‐function (LOF) mutations. Synonymous mutations do not alter protein sequences, whereas non‐synonymous and LOF mutations can affect protein structure or function.

For non‐synonymous SNPs, predictions of their effects on protein function were made using the SIFT4G (v1.0) (Vaser et al. 2016) and Grantham scoring systems (Grantham 1974). SIFT4G employs a statistical method to assess whether the amino acid substitutions affect protein function, with scores below 0.05 indicating potential deleterious effects. Grantham scores above 150, which measure amino acid substitution similarity, suggest significant chemical differences that likely affect protein function. To minimize prediction errors, allele frequencies were considered: alleles with frequencies above 50% were considered ancestral and assumed not to affect protein function, whereas alleles with frequencies below 50% were deemed derived and potentially deleterious. The derived alleles with potential deleterious effects were extracted and analyzed using vcftools.

To quantify the mutation load, we used specific metrics: for each mutation category (DEL and LOF), the proportion of deleterious mutations (ρa) was calculated as ρa = A/(2 × *N*_*total*_), where A represents the total number of SNPs in each category and *N*_*total*_ is the total number of SNPs annotated for deleterious mutations. Heterozygotes were counted once and homozygotes were counted twice to account for the potential amplified effect on the phenotype. We also computed the proportion of heterozygous and homozygous deleterious (ρg) as ρg = G/*N*_*total*_, where G represents the total number of heterozygous or homozygous deleterious alleles. These values (ρa and ρg) were calculated for each individual to assess differences in mutation load, and the Wilcoxon test was used to compare species and lineage differences. This nonparametric test is suitable for comparing the median differences between independent samples.

### 2.5 Identification of Genomic Islands of Divergence and Genes Under Positive Selection

To identify the genomic regions with high differentiation, per‐window *F*_ST_ values were computed for 50‐kb nonoverlapping windows, each containing varying numbers of variable sites. Subsequently, *Z*‐transformed *F*_ST_ values were calculated using the formula (Han et al. 2017): *Z*‐*F*_ST_ = (Per‐windows *F*_ST_ − Mean *F*_ST_)/Standard deviation of *F*_ST_. Outlier windows of “*F*_ST_‐islands” were pinpointed where *Z*‐*F*_ST_ ≥ 2. Finally, the adjacent windows with *F*_ST_‐islands were combined into a larger divergence region.

The XP‐CLR (v1.0) test was applied to detect selective sweeps within each 50‐kb nonoverlapping window across the genome (Chen, Patterson, and Reich 2010), comparing the Yili and Tacheng groups. Genes within *F*_ST_‐islands exhibiting the highest 5% XP‐CLR values were identified as putatively positively selected genes (PSGs). To further elucidate their functions, these PSGs were subjected to Gene Ontology (GO) enrichment analysis using DAVID program (v6.8) (Sherman et al. 2022). Gene Ontology terms with a false discovery rate (FDR) of < 0.05 were considered significantly enriched (Benjamini and Hochberg 1995).

## 3 Results

### 3.1 Genetic Structure

We conducted deep resequencing of the genomes of 40 individuals of *M. sieversii* and 13 individuals of *M. baccata* , achieving a 25× coverage depth. After rigorous data quality control and filtering, we identified 12,278,281 SNPs for genetic diversity estimation and analysis. We ensured that all of *M. sieversii* and *M. baccata* samples were free of genetic admixtures with *M. domestica* (Figure S1).

**FIGURE 2:**
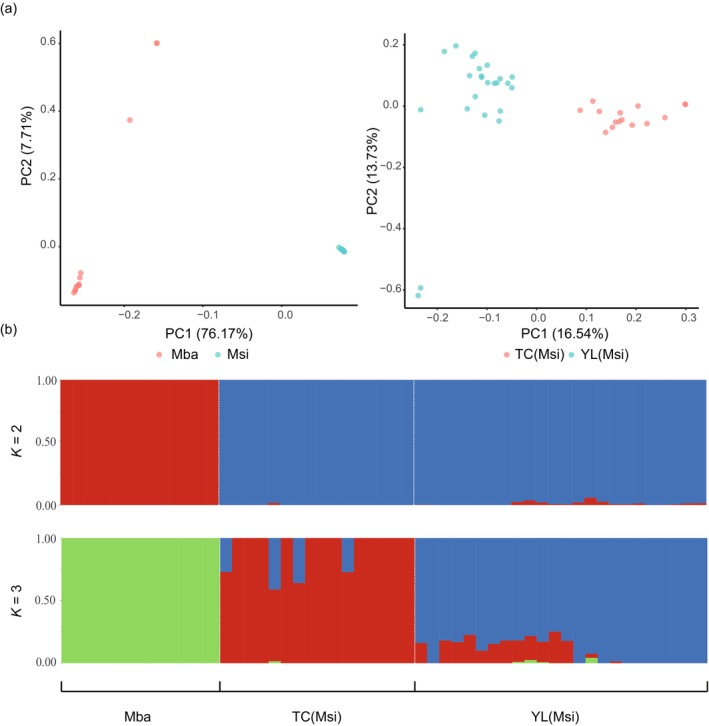
(a) Principal component analysis (PCA) showing the first two principal components of *Malus sieversii* and *M. baccata* ; (b) genetic clustering of these sampled individuals using ADMIXTURE at *K* = 2 and *K* = 3. Mba represents *M. baccata* , and Msi represents *M. sieversii* .

Using PCA and ML tree construction, we observed a significant genetic differentiation between *M. sieversii* and *M. baccata* , and within the *M. sieversii* (Figure 2). Specifically, *M. sieversii* populations were divided into two distinct lineages associated with their geographic distribution. One group included populations from the Yili region (GL, HC, and XY), and the other group included the populations from the Tacheng region (EM and TL) (Figure 2).

We conducted an ADMIXTURE clustering analysis on the SNP data to investigate the genetic structure of these groups after removing linkage disequilibrium. A distinct separation between the two species was observed in the clustering analysis, with *K* = 2. At *K* = 3, the clustering results aligned with both PCA and ML tree construction, further subdividing the *M. sieversii* population into two main lineages (Figure 2). In addition, the cross‐validation (CV) values at *K* = 2 and *K* = 3 were low, indicating more reliable results under these conditions.

### 3.2 Genetic Diversity

**FIGURE 3:**
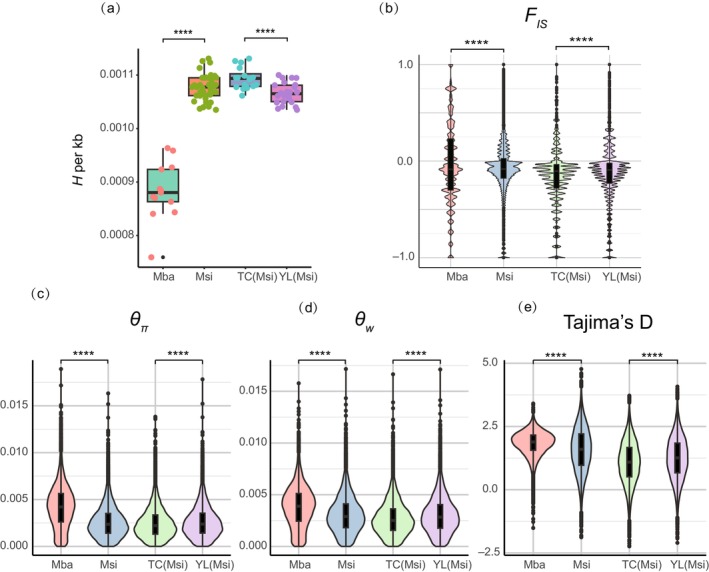
(a) Comparisons of the genome heterozygosity (*H*) between species and lineages; (b–e) distributions of nucleotide diversity parameters (*F*_IS_, *θπ*, *θW*, and Tajima's D) between species and lineages across the genome. *P*‐values for pairwise comparisons were calculated using the Wilcoxon test: ****p < 0.0001. Mba represents *M. baccata* , and Msi represents *M. sieversii* .

Significant differences in genetic diversity were observed between *M. baccata* (Mba) and *M. sieversii* (Msi) (Figure 3). Genetic diversity indices were initially calculated for both species and for the YL and TC lineages within *M. sieversii* . *M. sieversii* exhibited a significantly higher genomic heterozygosity than *M. baccata* .

Subsequent analysis of *M. sieversii* revealed that TC lineages displayed higher genomic heterozygosity than YL lineages. Nucleotide diversity (*θ*_*π*_) analysis indicated a higher value for *M. baccata* , suggesting greater genetic variation accumulation and richer genetic diversity in this species. Similarly, in *M. sieversii* , the YL lineage exhibited higher nucleotide diversity than the TC lineage, further supporting the higher genetic diversity of the YL lineage.

Watterson's estimator (*θ*_*W*_) was utilized to estimate the effective population size (*Ne*) based on nucleotide diversity. The *θ*_*W*_ value for *M. baccata* was higher than that of *M. sieversii* , indicating a larger effective population size in *M. baccata* . Within *M. sieversii* , the YL lineages had a higher *θ*_*W*_ compared to the TC lineages, consistent with nucleotide diversity results.

Tajima's D values indicate population expansion events in both species. *M. baccata* , which exhibited high genetic diversity, also displayed a high Tajima's D value, indicating a pronounced population expansion. Similarly, the YL lineages of *M. sieversii* , characterized by higher genetic diversity, demonstrated a higher Tajima's D value, suggesting increased population expansion events. *F*_IS_ values, which reflect within‐population kinship, were negative for both species, indicating some degree of inbreeding. Negative *F*_IS_ values in the TC and YL lineages of *M. sieversii* may indicate significant bottleneck events that contribute to inbreeding.

### 3.3 Demographical History

**FIGURE 4:**
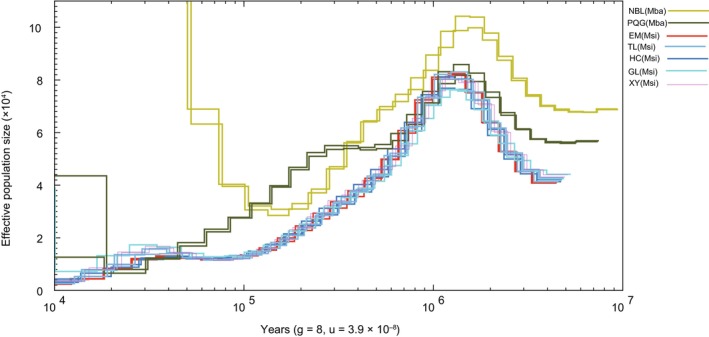
Population size over time plots for *Malus sieversii* and *M. baccata* based on pairwise sequential Markovian coalescent (PSMC) modeling. Mba represents *M. baccata* , and Msi represents *M. sieversii* .

Population history analyses of *M. baccata* and *M. sieversii* revealed distinct population dynamics across their evolutionary histories. *M. baccata* displayed an expansion–contraction–re‐expansion pattern, whereas *M. sieversii* showed an expansion–contraction pattern (Figure 4). In particular, at approximately 1 Ma, the population sizes of both *M. baccata* and *M. sieversii* experienced a bottleneck. Following this period, the NBL populations of *M. baccata* experienced substantial expansion, whereas the PQG population continued to contract until it expanded during the late Quaternary. *M. sieversii* populations exhibited a similar trend: *M. sieversii* populations continued to decline over the past million years.

### 3.4 Mutation Load and Mutation Accumulation

**FIGURE 5:**
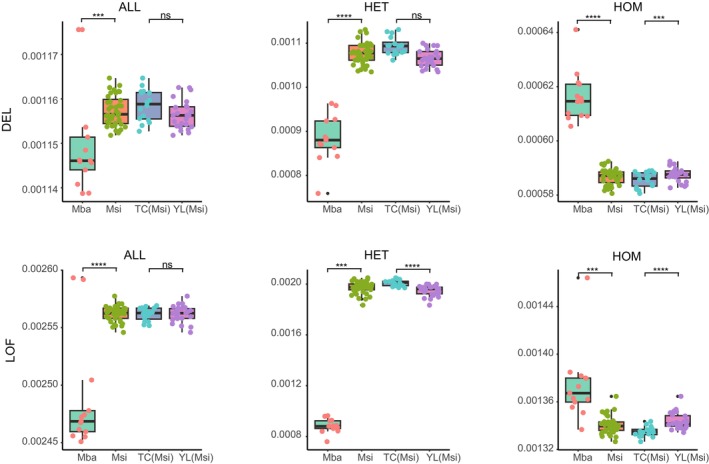
Accumulation and purging of deleterious mutations in *Malus sieversii* and *M. baccata* . Proportion of derived alleles (ALL), heterozygous genotypes (HET), and homozygous‐derived genotypes (HOM) in mildly (DEL) or putatively highly deleterious (LoF) mutations in *Malus sieversii* (Msi), *M. baccata* (Mba), and lineages within *M. sieversii* (TC(Msi), and YL(Msi)). *P*‐values for pairwise comparisons were calculated using the Wilcoxon test: ***p < 0.001; ****p < 0.0001; ns, not significant.

Analysis of the SNP data from *M. baccata* and *M. sieversii* revealed differences in the accumulation of deleterious mutations (Figure 5). For *M. baccata* , the number of SNPs ranged from 13,008,421 to 13,115,226 variants, with heterozygous counts ranging from 2,196,300 to 3,587,490, and homozygous counts from 9,499,989 to 10,918,926. In contrast, *M. sieversii* exhibited slightly higher SNP numbers, ranging from 13,207,668 to 13,214,172 variants, with heterozygous counts ranging from 2,075,037 to 2,387,899, and homozygous counts ranging from 10,823,538 to 11,136,212.

Regarding the accumulation of the deleterious mutations, *M. baccata* showed a lower accumulation of both mildly deleterious mutations (DEL) and LOF compared to *M. sieversii* . Specifically, the number of deleterious mutations in heterozygotes was significantly lower in *M. baccata* than in *M. sieversii* . However, *M. baccata* accumulates more deleterious mutations in homozygotes than does *M. sieversii* (Figure 5).

In *M. sieversii* , the YL and TC lineages exhibited similar trends. The TC lineages showed a slightly higher accumulation of DEL than the YL lineages, whereas the accumulation of LOF was similar in both lineages. For DEL accumulation in heterozygotes, the TC lineages showed higher accumulation than the YL lineages, although the difference was not statistically significant (*p* > 0.05). However, the TC lineages showed significantly higher LOF accumulation than the YL lineages (*p* < 0.0001).

In terms of homozygous DEL accumulation, the YL lineage had significantly higher accumulation than the TC lineage (*p* < 0.001). Similarly, for homozygous LOF accumulation, YL lineages accumulated significantly more than the TC lineages (*p* < 0.0001).

### 3.5 Genomic Islands of Divergence and Genes Under Positive Selection

**FIGURE 6:**
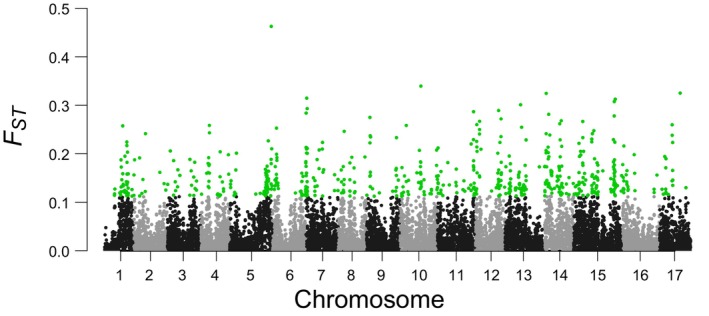
Manhattan plot of the population differentiation (FST) in 50 kb nonoverlapping windows. Alternating colors represent background windows on different chromosomes, and “*F*_ST_‐islands” are highlighted in green.

The genomic islands of divergence between the Yili Group and Tacheng Group were defined as regions with *Z*‐*F*_ST_ ≥ 2 compared to the genomic background. A total of 625 extreme 50‐kb outlier windows (4.67% of the genome) were detected. After merging adjacent outlier windows, 404 genomic islands were identified scattered across the genome (Figure 6). Among these chromosomes, Chr 11 harbored the highest number of outlier windows (13.8% of all outlier windows) when compared to other chromosomes.

To identify the genes undergoing positive selection within these genomic islands, we performed an XP‐CLR test between the Yili and Tacheng group genomes. A total of 767 PSGs were identified in these genomic islands. GO enrichment analysis of these PSGs revealed their involvement in ion transport processes (e.g., glutamate receptor 2.7‐like, mechanosensitive ion channel protein 10‐like, potassium channel KAT1‐like), protein phosphatase activities (e.g., protein phosphatase 2C 70‐like, putative dual specificity protein phosphatase DSP8), ion transmembrane transport (e.g., aluminum‐activated malate transporter 12‐like, aluminum‐activated malate transporter 13‐like, aluminum‐activated malate transporter 8‐like), malate transport (e.g., aluminum‐activated malate transporter 12‐like, aluminum‐activated malate transporter 13‐like, aluminum‐activated malate transporter 8‐like), mitochondrion organization (e.g., clustered mitochondria protein, mitochondrial Rho GTPase 2‐like, nuclear intron maturase 1, mitochondrial), and plasma membrane functions (e.g., abscisic acid receptor, calcineurin B‐like protein 10, probable magnesium transporter NIPA6).

## 4 Discussion

### 4.1 Evolutionary History of Endangered *M. sieversii*

In this study, we identified two distinct lineages among the five Chinese populations of *M. sieversii* : the Yili and Tacheng lineages (Figure 2). This genetic differentiation was consistent with the findings of various molecular marker studies (Zhang, Li, and Li 2018). Prior research has indicated that the Tacheng lineage, comprising populations EM and TL, exhibits a unique genetic structure compared to other populations throughout the entire distribution range of *M. sieversii* (Zhang et al. 2021). We determined that the Tacheng lineage diverged from the Yili lineage during the late Miocene (approximately 5.38 million years ago) (Zhang et al. 2021), likely owing to aridification in the Tianshan Mountains. Following prolonged geographic isolation, the Tacheng lineage developed a significantly higher level of genetic differentiation than the Yili lineage. We identified a total of 404 genomic islands across the genome (Figure 6), of which 767 PSGs were annotated and associated with physiological processes such as ion transport, ion transmembrane transport, malate transport, and plasma membrane functions. This suggests that different habitats (Zhang, Li, and Li 2018) facilitated physiological adaptations between the Yili and Tacheng lineages.

The demographic history of *M. sieversii* indicates that it underwent a bottleneck effect at approximately 1 Ma (Figure 4). This event coincided with the peak of glacial periods in the Tianshan Mountains (Xu et al. 2010), characterized by prevailing cold and dry climates that promoted the bottleneck effect in *M. sieversii* . Similarly, the widely distributed species *M. baccata* experienced a bottleneck effect at approximately 1 Ma (Figure 4). However, while the effective population size of *M. baccata* recovered between 100,000 and 10,000 years ago (Figure 4), that of *M. sieversii* has continued to decline over the past million years. This ongoing reduction in effective population size may ultimately lead to the endangered status of *M. sieversii* . Similar patterns have been observed in other species pairs, such as *Ostrya* and *Dipteronia*, where endangered species showed a decline in effective population size compared to their widespread congeners (Yang et al. 2018; Feng et al. 2024).

### 4.2 Genomic Insights Into Endangerment of *M. sieversii*

The endangered status of *M. sieversii* and its limited distribution is closely linked to environmental changes throughout its evolutionary history. In contrast, *M. baccata* , which thrives in more stable and humid conditions, exhibits a wider distribution across northern China and Northeastern Asia (Figure 1). Significant genetic differences between the two species have been observed (Figure 2), highlighting the influence of their distinct habitats. Wang et al. (2024) emphasized the critical role of environmental conditions in shaping the genetic diversity of these species. Demographic history analysis suggests that the effective population size of *M. sieversii* has steadily declined over the past million years, likely due to increasingly arid habitat conditions (Figure 4). Conversely, *M. baccata* experienced population recovery between 100,000 and 10,000 years ago, which has contributed to its broader distribution. Wang and Li (2018) reported similar findings, which underscores the significance of population dynamics in species conservation. Furthermore, genetic diversity indices indicate that species with broader ranges, such as *M. baccata* , exhibit higher levels of genetic diversity compared to those with narrower ranges (Figure 3c). This observation aligns with the hypothesis that species with wider distributions retain more genetic alleles. Additionally, due to ongoing habitat loss and declining effective population size, *M. sieversii* has accumulated a greater number of deleterious mutations than *M. baccata* (Figure 5), as discussed by Wang et al. (2024).

Within *M. sieversii* , the current distribution area of the Yili lineage is approximately five times larger than that of the Tacheng lineage (Yan and Xu 2010). We sampled three populations from the Yili lineage, and two populations from the Tacheng lineage (Figure 1). The Yili lineage exhibited an isolated genetic structure from the Tacheng lineage (Figure 2), which is consistent with previous studies (Zhang, Li, and Li 2018; Zhang et al. 2021). In response to regional climatic changes in the Tianshan Mountains, both the Yili and Tacheng lineages shared similar demographic histories (Figure 4). They experienced a decline in effective population size following the larger stage of glacial periods in the Tianshan Mountains (Xu et al. 2010). This suggests that *M. sieversii* has continuously lost suitable habitats and experienced declining effective population sizes throughout its evolutionary history. Similar phenomena have been observed in endangered species such as *Dipteronia dyeriana* and *Ostrya rehderiana*, including the fixation of strongly deleterious mutations, reduced fitness, and continuous population decline (Yang et al. 2018; Feng et al. 2024).

### 4.3 Conservation Managements

*Malus sieversii* is classified as a second‐ranked conservation priority in the China Plant Red Data Book (Fu 1992). This species is one of the ancestors of the domesticated apple trees (Cornille, Gladieux, and Giraud 2013). Previous studies identified priority populations based on indices of genetic diversity (Zhang, Li, and Li 2018). In this study, the Yili lineage exhibited a level of genetic diversity similar to that of the Tacheng lineage (Figure 3). However, significant genetic divergence was observed between these two lineages (Figure 2). Across the entire distribution range of *M. sieversii* , the Tacheng lineage also displays distinct genetic clustering compared to other populations (Zhang et al. 2021). Our observations have important implications for understanding the conservation management of *M. sieversii* . To preserve the integrality of the genetic resources, we recommend the establishment of separate conservation units for the Yili and Tacheng lineages. Throughout the evolutionary history of *M. sieversii* , we observed a decline in effective population size over the past million years (Figure 4). Our results highlight that the Tacheng lineage has a much smaller distribution range and exhibits a higher frequency of deleterious mutations than the Yili lineage. The Tacheng lineage faces greater challenges due to its climatic and geographical conditions, which contribute to the accumulation of deleterious mutations within its populations. Therefore, conservation management for Tacheng lineage should be aimed at prioritizing the preservation of germplasm resources and the mitigation of its unique geographical challenges. This approach includes the creation of gene banks to conserve genetic resources, the introduction of new individuals to enhance population diversity, and the improvement of habitat quality through ecological restoration initiatives. In contrast, Yili lineage exhibits favorable climatic conditions for the growth of *M. sieversii* and a lower frequency of deleterious mutations, but many of their distribution areas have experienced anthropogenic disturbances. Therefore, conservation efforts for Yili lineage should implement measures to restore and protect its habitat. Specifically, the establishment of nature reserves, the execution of ecological restoration projects, and the reduction of human interference will be critical for the conservation of Yili lineage. By implementing these tailored conservation strategies in Yili and Tacheng, we can effectively address the specific environmental and population characteristics of each region, thereby ensuring the long‐term survival and development of *M. sieversii* .

## Conflicts of Interest

The authors declare no conflicts of interest.

## Supporting information


Figures S1–S2

## Data Availability

The newly obtained raw resequencing reads were available on the National Genomics Data Center (https://ngdc.cncb.ac.cn/) Genome Sequence Archive (GSA) under accession number CRA020232.
