# Supplementary material for: Evolutionary Genomics Provides Insights Into Endangerment and Conservation of a Wild Apple Tree Species, Malus sieversii
Source: Evol Appl. 2024 Dec 4;17(12):e70048. doi: 10.1111/eva.70048 (PMC11616530; doi:10.1111/eva.70048)
Supplement: Supplementary file 1 — Figures S1–S2 [file EVA-17-e70048-s001.doc]

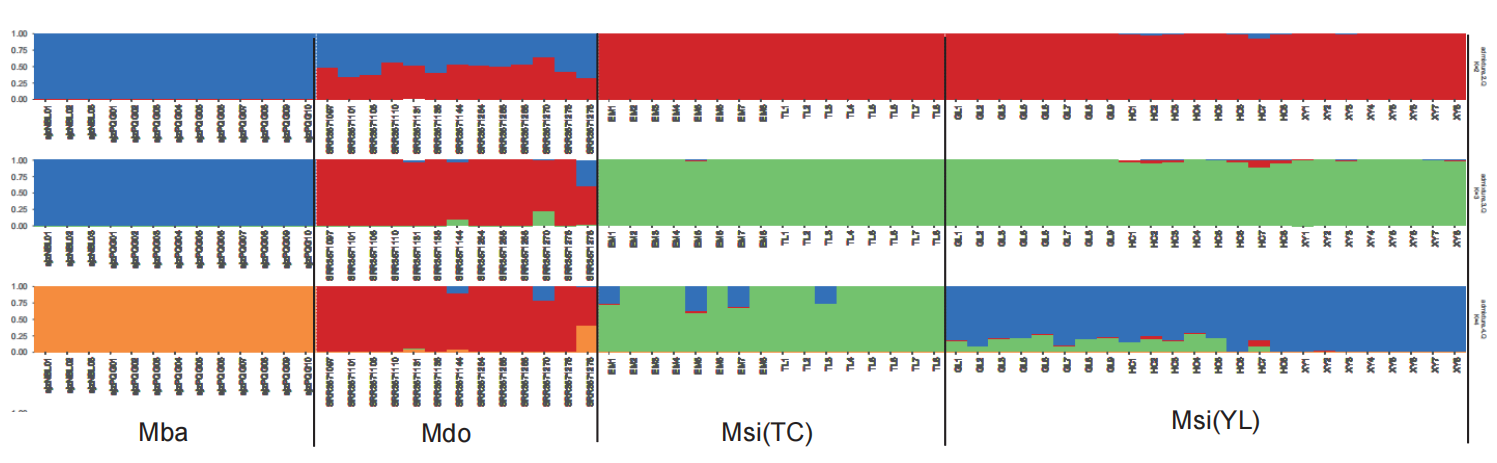


Fig. S1 Genetic clustering of these sampled individuals using ADMIXTURE at K = 2, K=3 and K=4. Mba represents *M. baccata*, Mdo represents *M. domestica*, and Msi represents *M. sieversii*.


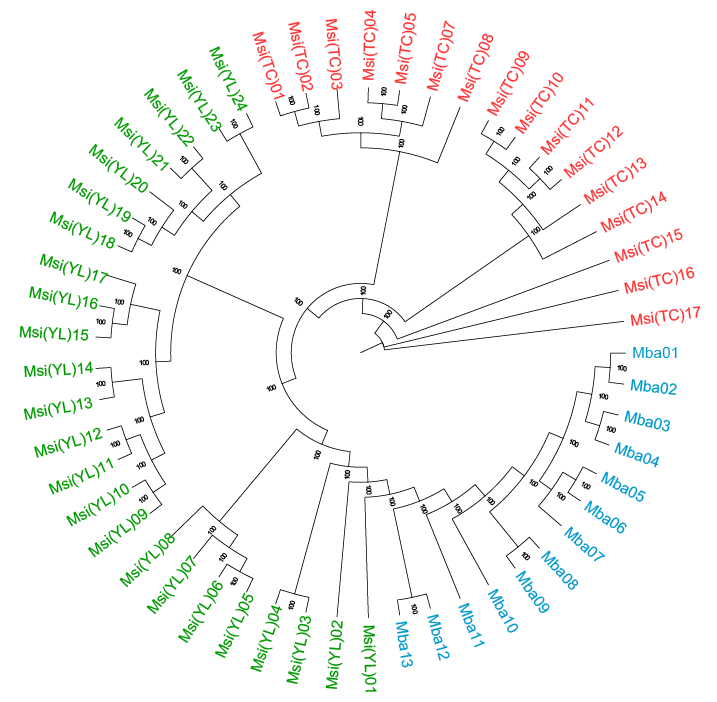


Fig. S2 Maximum-Likelihood (ML) phylogenetic tree of these sampled individuals of *M. baccata* and *M. sieversii*. The numbers near the branches show the bootstrap values of the nodes (%).Mba represents *M. baccata*, Msi represents *M. sieversii*.
